# Efficacy of Anakinra for Various Types of Crystal-Induced Arthritis in Complex Hospitalized Patients: A Case Series and Review of the Literature

**DOI:** 10.1155/2015/792173

**Published:** 2015-03-26

**Authors:** A. Aouba, S. Deshayes, L. Frenzel, A. Decottignies, C. Pressiat, B. Bienvenu, F. Boue, G. Damaj, O. Hermine, S. Georgin-Lavialle

**Affiliations:** ^1^Department of Internal Medicine, CHU de Caen, Université de Caen, Basse Normandie, avenue de la Côte de Nacre, Caen, France; ^2^Department of Haematology, Hôpital Necker-Enfants Malades, 149 rue de Sevres, 75015 Paris, France; ^3^Department of Pharmacy, Hôpital Antoine Béclère, 157 rue de la Porte de Trivaux, 92141 Clamart Cedex, France; ^4^Department Haematology, CHU de Caen, Université de Caen, Basse Normandie, avenue de la Côte de Nacre, Caen, France; ^5^Department of Internal Medicine, Hôpital Tenon, Université Paris 6 Pierre et Marie Curie, DHU I2B, 4 rue de la Chine, 75020 Paris, France

## Abstract

*Background*. There are few data on anakinra use after failure of conventional medications for crystal-induced peripheral arthritis and/or crowned dens syndrome among complex hospitalized patients. *Methods*. We retrospectively analyzed the outcome of six patients affected with subacute crystal-induced arthritis who had received anakinra in second or third line therapy, including three patients with crowned dens syndrome and three others with gouty arthritis. Patients' comorbidities, reasons for anakinra use and associated drugs, and outcomes were recorded. *Results*. All patients presented with elevated inflammatory syndrome, systemic symptoms with poly/oligoarthritis. Except for absolute contraindications, all patients were previously treated with full or decreased dose of NSAID, colchicine, and/or glucocorticoids, with unsatisfactory response. All three gouty patients exhibited complete responses in all acute involvements under anakinra within 3 to 5 days, including one of them who needed the reintroduction of colchicine treatment that was previously unsuccessful. Crowned dens syndrome patients, including two with pseudogout and one with subacute hydroxyapatite deposition disease, needed 9 to 11 days to achieve complete response. Tolerance to anakinra was good. *Conclusion*. In case series of complex hospitalized patients, anakinra showed good activity in crowned dens syndrome and associated crystal-induced peripheral arthritis, with longer treatment duration than in gouty arthritis.

## 1. Introduction

The number of therapeutic drugs used to treat crystal-induced arthritis (CRIA) is relatively limited, relying on colchicine, nonsteroidal anti-inflammatory drugs, and corticosteroids. These drugs may have significant and severe side-effects or may be contraindicated and therefore cannot always be used at an optimal dosage especially among elderly patients. Thus, other therapeutic options are often required in clinical practice.

Recently, several publications have focused on the rationale and efficacy of the interleukin-1 blockade mainly in gout [1–4] and sparsely in pseudogout [5–7]. No clear outcome parameters concerning notably the treatment duration of anakinra are defined. Otherwise, there is no report on the use of anakinra in the acute inflammatory manifestations of hydroxyapatite deposition disease (HADD) and notably in crowned dens syndrome, which is a clinical form that this latter shares with pseudogout. Both diagnosis and management of crowned dens syndrome can be challenging, and the control of the disease requires sometimes the combination of several drugs, with increased risk of toxicity especially in older patients.

Herein, we report on the successful use of anakinra in the context of resistance or contraindications to conventional drugs, in six complex hospitalized elderly patients with crowned dens syndrome and associated crystal-induced peripheral arthritis (*n* = 3) and gouty polyarthritis on the other hand (*n* = 3).

## 2. Patients and Methods

We retrospectively collected data from six adult patients who had received off-label anakinra (Kineret 100 mg/day; subcutaneous injection) for CRIA (gout, pseudogout, or HADD) between January 2012 and March 2013. All patients included in this study were hospitalized or referred to the department of internal medicine. Approvals from institutional medical-pharmaceutical committee and review board were obtained for this study, and the patients' consent was obtained after providing them with information about anakinra.

The diagnosis of gout was based on the presence of monosodium urate (MSU) crystals in synovial fluid, the presence of tophus, or the presence of at least six of the twelve other crystal-induced types of arthritis referred to in the criteria of the American College of Rheumatology [8]. The diagnosis of other types of CRIA was based on the same scheme, except for the characteristic signs of gout, including hydroxyapatite crystals for HADD or calcium pyrophosphate (CPP) crystals for pseudogout.

In the setting of complex hospitalized patients, diagnosis of an attack of CRIA was based on the presence of compatible clinical signs, significant increase in C-reactive protein levels (CRP; normal < 5 mg/L) to at least 15 mg/L, and associated typical imaging. Some differential diagnoses were ruled out by performing minimal autoimmune (antinuclear factor, anti-DNA, rheumatoid factor, anti-CCP, and ANCA antibodies) and routine microbiological exams. Serum uric acid levels were assessed (>6.0 mg/dL in women) also taking account of creatinine level. None of our six patients had a medical history or other clinical manifestations that evoked an inherited autoinflammatory syndrome.

For an attack of CRIA, colchicine was given to patients 1 and 6, at 1 mg/three times a day, on days 1 and 2; then 1 mg twice daily, on days 3 and 4; and then 1 mg a day on days 5 and 6. Reduced doses, for best tolerance with similar efficacy [2], were given to patients 2 and 4, whereas this drug was contraindicated in patients 3 and 5 (Table 1). Long-term prophylaxis was given to patients who had exhibited previous recurrent attacks of polyarthritis with respect to renal function [2]. The nonsteroidal anti-inflammatory drugs that were routinely used were indomethacin (orally, 25 mg, 3-4 times daily) or ketoprofen (100 mg twice daily, orally and/or intravenously) given for 10–14 days; doses were reduced by 30% for elderly patients and those at risk of renal damage with close creatinine monitoring. Prednisone was given at 0.5 mg/kg/day (max. 40 mg/day) for 15 days. Anakinra therapy was maintained until there was both a complete clinical response and normalization of CRP level. Treatment with anakinra was stopped by day 6 in the absence of any clinical improvement and no reduction in CRP level, which was monitored every 48–72 h.

## 3. Results

### 3.1. Main Features of the Patients

**Figure 1:**
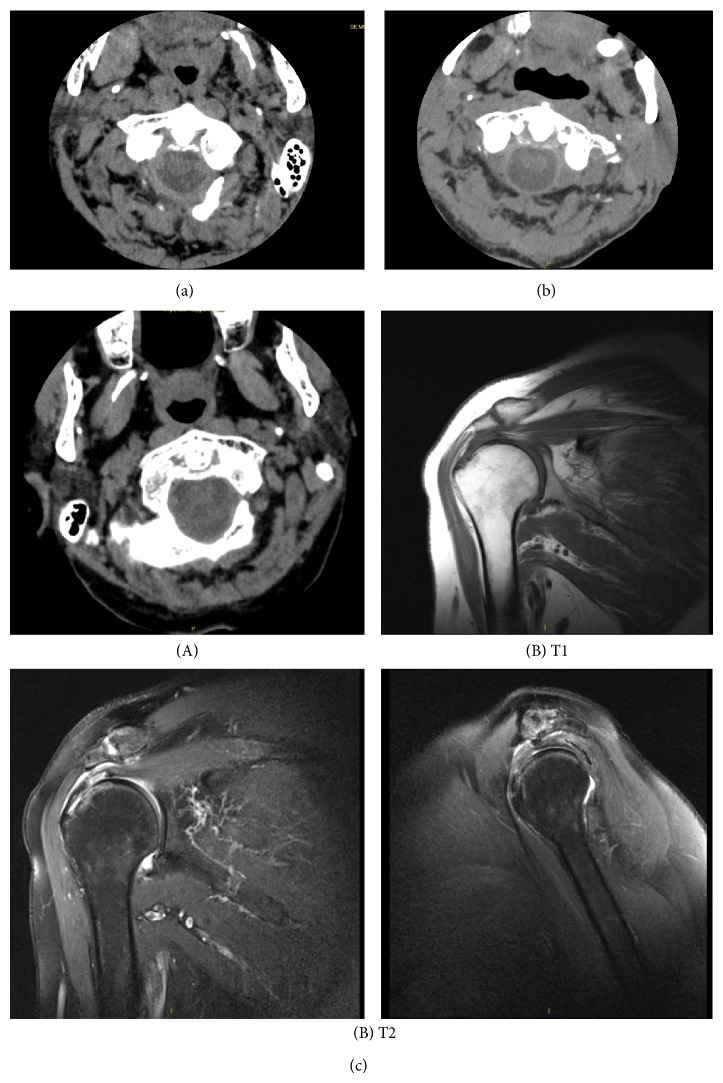
Iconographic features of crowned dens syndrome and crystal-induced peripheral arthritis of patient 1 (a), patient 2 (b), and patient 3 (c(A) and c(B)). (a) Cervical CT-scan centered on the odontoid process: fine linear arciform calcification of the retroodontoid ligament suggesting pseudogout involvement. (b) Cervical CT-scan centered on odontoid process: cloudlike calcification of the retroodontoid ligament suggesting hydroxyapatite disease deposition involvement. (c(A)) Cervical CT-scan centered on odontoid process: thick arciform calcification of the retrodens ligament. (c(B)) Right shoulder MRI (T2 and T1): supraspinatus tendinopathy with partial rupture aspect. Tenosynovitis of the long biceps. Tendinopathy of the infraspinatus and subscapularis tendons.

Six patients were included in the study: three patients with crowned dens syndrome and associated acute/subacute crystal-induced peripheral arthritis (patients 1, 2, and 3; Table 1; Figure 1) and three other with gout (patients 4, 5, and 6; Table 1). They were two men and four women with a mean age of 72 years, ranging from 65 to 88. Most of them displayed comorbidities such as arterial hypertension (*n* = 4), hypothyroidism (*n* = 2), chronic kidney disease (*n* = 2), obesity (*n* = 1), diabetes (*n* = 1), and chronic hematological malignancies (MGUS: *n* = 1, prolymphocytic leukemia: *n* = 1, and polycythemia vera: *n* = 1). All patients exhibited low grade fever, alteration of the general status, and marked and/or persistent increased CRP levels over 3 weeks (Table 1).

Patients with crowned dens syndrome were more likely aged females (three women with mean age of 79 years) compared to gouty patients (two men, one woman with mean age of 69.6 years). For the three patients with crowned dens syndrome, the diagnosis was retained on clinical signs, namely, fever, multidirectional neck stiffness and pain, exclusion of meningitis with lumbar picture in two, and compatible features of neck CT-scan without any criteria for vertebral or soft-tissue infectious or tumoral process (Figure 1). The diagnosis of crowned dens syndrome was moreover based on concomitant peripheral joint involvements criteria (Table 1). Two patients had, respectively, a history of pseudogout and HADD diagnosed on typical crystal finding on joint puncture and compatible X-ray features. The latter exhibited calcium pyrophosphate crystals on knee joint puncture and iconographic pictures of pseudogout location, but also thick arciform calcification of the retrodens ligament on cervical CT-scan and HADD features on scapular MRI (Figures 1(c)(A) and 1(c)(B)).

Among the three gouty patients, two were treated, respectively, with hydroxyurea and allopurinol for polycythemia vera and with pentostatin for a prolymphocytic leukemia without exhibiting criteria for tumor lysis syndrome; the latter exhibited severe chronic renal insufficiency related to gouty nephropathy.

The localization of arthritis was different in the two groups: gouty patients presented symmetrical large and small joints arthritis whereas patients with crowned dens syndrome displayed by definition a periodontoid arthritis and often asymmetrical shoulder and/or wrist involvements.

### 3.2. Anakinra Efficacy among Patients with Crowned Dens Syndrome and Gout

Indication for anakinra was mostly similar for both groups, including failure or contraindications to colchicine and/or nonsteroidal anti-inflammatory drugs or corticosteroids, especially for the associated comorbidities.

**Figure 2:**
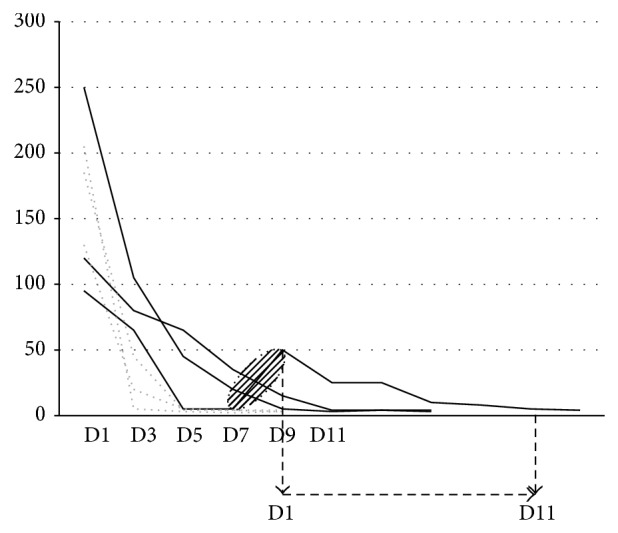
Evolution of CRP levels on gouty polyarthritis patients (*n* = 3) and crowned dens patients associated with peripheral pseudogout or hydroxyapatite deposition disease patients (*n* = 3) arthritis under anakinra. Normalization of CRP levels at the 3rd or 5th days for the three polyarthritis gouty patients (grey and dotted lines) associated with complete clinical response and stopping anakinra. Same but later upcoming for two crowned dens syndrome patients (black continue lines), respectively, at the 9th or 11th days of treatment. For the 3rd crowned dens syndrome patient, early cessation of the treatment stopping within the 7–9th days (hatched ellipse) showed reincrease of CRP levels; resuming of anakinra for 11 days showed complete and sustained control of CRP levels and clinical signs.

Complete remission, defined both clinically and biologically with the normalization of CRP level (Figure 2), was obtained in all three gouty patients, whereas for the crowned dens syndrome group only two of three patients underwent complete remission with only anakinra. In the crowned dens syndrome group (patients 1, 2, and 3; Table 1), 9 and 11 days were, respectively, needed to induce complete remission for the two responders, whereas in gout only a median of 5 days (3, 5, and 5 days, resp.) was needed. For the third patient with crowned dens syndrome, anakinra withdrawal on the 7th day in the context of marked reduction but persistent neck pains was followed by a rapid disease flare-up 48 h later; the reintroduction of a second course of anakinra led to rapid, complete, and persistent remission of all acute clinical signs associated with CRP level normalization at the 11th day, allowing for withdrawing the treatment this same day. Finally, a median of 11 days (9, 11, and 11 days, resp.) was required for complete remission in crowned dens syndrome patients.

Finally, a gouty patient (patient 6) exhibited a relapse six weeks after anakinra withdrawal. While the reintroduction of anakinra did not allow disease control at the 5th day, adjunction of colchicine led to persistent complete remission at the 3rd day of the combined treatment. For all six patients, after anakinra withdrawal, dietary recommendations associated with allopurinol, febuxostat, and/or colchicine were continued as prophylactic measures; no further relapse occurred within a follow-up of 6 to 16 months.

### 3.3. Treatment Safety

Anakinra showed good or excellent tolerance profiles among all patients. Two patients (patients 1 and 4) had mild injection-site reactions and transient diffuse pruritus. No episodes of neutropenia or infection were observed. Despite initial decrease in renal function (creatinine clearance: 40–20 mL/min) in some patients, daily injections of anakinra were not harmful and even improved the renal parameters of patient 4 who probably had chronic gouty nephropathy. Colchicine tolerance was also good, although patients 2 and 4 presented episodic diarrhea. Finally, for patient 3 who had an ongoing staphylococcal soft-tissue infection, the use of anakinra rather than colchicine was harmless.

## 4. Discussion

In the context of failure or contraindications to conventional therapies, anakinra exhibited good efficacy in all three types of CRIA with a longer treatment duration in crowned dens syndrome and associated peripheral arthritis related to pseudogout and/or HADD (9, 11, and 11 days, resp.) than in gouty arthritis (3, 5, and 5 days, resp.). Moreover, adjunction of colchicine to anakinra seems to allow a best outcome in CRIA refractory to each drug alone. To the best of our knowledge, this is the first report of anakinra use and efficacy for crowned dens syndrome related to pseudogout and HADD and also for HADD peripheral subacute arthritis. The tolerance in elderly patients was good in our series. Indeed, several articles showed that anti-IL-1 agents, such as anakinra, are safe in comparison with other biotherapies, such as anti-TNF-alpha, considering the risk of tuberculosis reactivation [9, 10].

Only few small series have been reported on the use of anakinra in gout and pseudogout as curative and/or prophylactic treatment. Moltó et al. showed that, in four out of five patients suffering from refractory subacute oligo/polyarticular flare-ups of pseudogout, anakinra use for three days allowed dramatic clinical control and normalization of CRP as soon as the third day [6]. Announ et al. and McGonagle et al. showed similar results for anakinra, used as a curative or prophylactic treatment for recurrent subacute or chronic polyarticular pseudogout in two patients for at least 6 months [5, 11]. Although Couderc et al. reported three patients with a satisfactory result with anakinra to prevent acute flare-ups, this drug was unable to control chronic synovitis symptoms in two of them [7].

Whereas gouty acute/subacute arthritis usually needed only three days of anakinra treatment, a longer treatment duration was necessary to control acute pseudogout flare-ups (14 days for the patient reported by McGonagle et al.) or chronic synovitis (i.e., at least three months), as observed for our patients and those in the literature [5, 7]. For two pseudogout polyarthritis patients, reported by Verhoeven et al., anakinra prescribed for five days showed no or only slight response [12]. These authors concluded that this poor efficacy of anakinra might be related to the systemic inflammation and/or to the long duration of this disease (2–6 months); these data also correlate with those of Couderc et al. [7].

Considering our pseudogout patients and those reported by others, this hypothesis appears only partially consistent. Treatment duration of five days leading to poor efficacy of anakinra in the series of Verhoeven et al. [12] was much shorter than the minimum of nine days in our series and for the patient reported by McGonagle et al. [5]. Thus, we hypothesize that treatment duration is the major factor in anakinra success to treat pseudogout and probably also HADD attacks. Indeed, in the pathophysiology of CRIA, the key role of IL-1 is now well documented: induction of IL-6 and TNF-alpha upregulation, complex retroinduction of other cytokines, leading to self-sustaining multicytokine vicious circle notably in cases of prolonged attacks [13–15].

Deposition of hydroxyapatite crystals in joints and periarticular tissues can cause acute attacks, qualified as pseudopodagra, which mimic gout and/or pseudogout attacks [13, 16]. Pazár et al. have shown that, similar to MSU and CPP, hydroxyapatite crystals can also act as alarmins and trigger NLRP3 inflammasome activation [13]. The consequent overproduction of IL-1*β* seems indirectly mediated by increased levels of other proinflammatory stimuli, including TNF-*α* or uric acid [17, 18]. Pazár et al. thus suggested that the IL-1 blockade may be clinically useful in acute HADD manifestations; this hypothesis was clinically confirmed for two of our patients [13]. Adding anakinra to previous ongoing treatments and, notably, colchicine should lead to the best response, as seen in the report of McGonagle et al. and for one of our polyarticular gouty patients [5].

To conclude, IL-1 blockade seems to be an interesting therapeutic option for all three types of CRIA whatever the type of joint involvement, including the debilitating and often misdiagnosed crowned dens syndrome. As observed in this short series and in sparse literature data, acute/subacute pseudogout or acute hydroxyapatite deposition arthritis should require extended duration of treatment with anakinra than in gouty arthritis [15]. Moreover, adding anakinra to conventional drugs could show better efficacy than each drug alone. Therefore, larger studies are needed to best assess the treatment modalities of anakinra in acute/subacute manifestations of the various types of CRIA, including its association with conventional drugs in curative and prophylactic purposes.

## Figures and Tables

**Table 1:** Main clinical features and outcomes of patients displaying crystal-induced arthritis treated with anakinra.

Patients	Crowned dens syndrome and associated peripheral crystal-induced arthritis			Polyarticular gout		
	Patient 1	Patient 2	Patient 3	Patient 4	Patient 5	Patient 6
Age/gender	65/F	88/F	84/F	66/M	71/M	72/F

Background condition	Pseudogout	Hydroxyapatite deposition disease (HADD)	Pseudogout (associated with HADD iconographic picture)	Gout with tophus	Gout	Gout

Comorbid conditions	Hypothyroidism	Essential arterial hypertension; hypothyroidism	Suspected Horton disease, postsurgical staphylococcal infected ingrown nail	Essential arterial hypertension; severe chronic renal insufficiency	Diabetes; grade 4 neutropenia to colchicine; polycythemia vera treated with hydroxyurea and allopurinol	Obesity; essential arterial hypertension; mild renal insufficiency; MGUS; allergy to allopurinol; prolymphocytic leukemia treated with pentostatin

Diagnosis	Crowned dens syndrome Pseudorheumatoid arthritis	Crowned dens syndrome Intra-articular acute HADD	Crowned dens syndrome, temporomandibular and peripheral pseudogout and/or HADD oligoarthritis	Subintrant articular gouty attacks Gouty nephropathy	Subintrant articular gouty attacks	Persistent articular gouty attacks

Acute joint involvement	Periodontoid structures Symmetrical large and small joints	Periodontoid structures Right shoulder Left wrist	Periodontoid and left temporomandibular joint structures, right shoulder and left wrist	Symmetrical large and small joints	Large and small joints	symmetrical large and small joints

Systemic signs (C reactive protein: CRP N < 5 mg/L)	Fever Anorexia Marked inflammatory syndrome (CRP ≥ 90 mg/L over 3 weeks)	Alteration of the general state Fever Elevated inflammatory syndrome (CRP ≥ 60 mg/L over 5 weeks)	Fever Alteration of the general state Diffuse headaches Marked inflammatory syndrome (CRP ≥ 210 mg/L over six weeks)	Alteration of the general state Fever Elevated inflammatory syndrome (CRP ≥ 150 mg/L over four weeks) Increase in creatininemia	Febricula Alteration of the general state Persistent inflammatory syndrome (CRP ≥ 85 mg/L over three weeks)	Febricula Alteration of the general state Inflammatory syndrome (CRP ≥ 65 mg/L over six weeks)

Indications for anakinra	Failure of ketoprofen, then colchicine and associated oral cortisone	Failure of colchicine and combined indomethacin, and oral cortisone	Ongoing staphylococcal soft tissues infection treated with rifampicin and vancomycin leading to avoiding colchicine and high dose steroid drugs	Failure of colchicine and associated oral cortisone	Contraindication to colchicine Failure of cortisone and indomethacin	Contraindication to allopurinol Failure of cortisone, indomethacin, and colchicine

Anakinra first treatment						
Duration	9 days	7 days	11 days	5 days	5 days	3 days
Associated drugs	None	None	Oral prednisone increased from 5 to 10 mg/day	None	None	None
Response	Complete remission of all acute clinical and biological parameters	Progressive improvement of clinical signs with slight cervical signs and normalization of CRP, allowing for consideration of complete response	Dramatic and complete response on the wrist and temporomandibular joints, progressive complete response on the crowned dens syndrome and the wrist involvement, normalization of CRP levels on the 15th day	Dramatic and complete remission of all acute clinical and biological parameters, and decrease in creatininemia	Rapidand complete remission of all acute clinical and biological parameters	Complete remission of all acute clinical signs, partial remission of biological parameters
Relapse or rebound	None, under prophylactic treatment of colchicine	Rebound 48 h after improvement at anakinra withdrawal on the 7th day	None, under prophylactic treatment with colchicine	None, under prophylactic treatment with colchicine and allopurinol	None,under prophylactic treatment with allopurinol	Disease relapse 6 weeks after the initial complete response

Anakinra second treatment						
Duration	NA	11 days, until complete response	NA	NA	NA	5 days
Associated drugs	NA	None	NA	NA	NA	None
Response	NA	Dramatic complete remission of all acute clinical and biological parameters	NA	NA	NA	Partial clinical and biological response
Relapse	NA	None, with prophylactic treatment with colchicine	NA	NA	NA	NA

Anakinra second treatment reintroduction						
Duration	NA	NA	NA	NA	NA	3 days
Associated drugs	NA	NA	NA	NA	NA	Colchicine
Response	NA	NA	NA	NA	NA	Complete clinical and biologic response, respectively, after 3 and 5 days of the combined treatment, respectively
